# The Disease Burden and Clinical Characteristics of Inflammatory Bowel Disease in the Chinese Population: A Systematic Review and Meta-Analysis

**DOI:** 10.3390/ijerph14030238

**Published:** 2017-02-28

**Authors:** Xue Li, Peige Song, Jun Li, Yuchang Tao, Guowei Li, Xiumin Li, Zengli Yu

**Affiliations:** 1 School of Public Health, Xinxiang Medical University, Xinxiang 453003, China; xue.li@ed.ac.uk (X.L.); p.song@sms.ed.ac.uk (P.S.); yuyuzaijia@163.com (Y.T.); 2 Centre for Population Health Sciences, University of Edinburgh, Edinburgh EH8 9AG, UK; 3 The 153 Hospital of People’s Liberation Army, Zhengzhou 450001, China; shuaidexue@126.com; 4 Department of Clinical Epidemiology & Biostatistics, McMaster University, Hamilton, ON L8S 4L8, Canada; lig28@mcmaster.ca; 5 Department Gastroenterology, the First Affiliated Hospital of Xinxiang Medical University, Xinxiang 453003, China

**Keywords:** epidemiology, phenotypes, Crohn’s disease, ulcerative colitis, inflammatory bowel disease

## Abstract

The temporal trend of inflammatory bowel disease (IBD) incidence is reported to be increasing in worldwide regions; however, reports focusing on China are sparse. The aim of this study was to provide an overview of the disease burden and clinical features of IBD in the Chinese population. We searched Medline, EMBASE, and another two Chinese databases. A parallel literature review and data extraction were conducted. Meta-analysis was performed to estimate the summary incidence rate of Crohn’s disease (CD) and ulcerative colitis (UC). The constituent ratios with 95% CI were calculated for clinical phenotypes and classifications. The literature review included 47 publications. The summary incidence rate of IBD was 1.74 (95% CI: 1.08; 2.40) per 100,000 person years, and the corresponding incidence rates of CD and UC were 0.40 (95% CI: 0.23; 0.57) and 1.18 (95% CI: 0.81; 1.56) per 100,000 person years, respectively. The sex distribution analysis indicated a male predominance in both CD (sex ratio: 1.64; 95% CI: 1.47–1.84) and UC (sex ratio: 1.29; 95% CI: 1.21–1.38). The clinical characteristics were summarized using data from 2283 CD cases and 17,958 UC cases; in which the majority of CD patients were diagnosed between 17–40 years of age, with non-stricturing and non-penetrating disease, varied disease locations, and less extra-intestinal manifestation. UC cases were featured with later disease diagnosis, a more severe disease course, more segmental lesions, and less extra-intestinal manifestations. Our study provided an estimated disease burden of IBD and demonstrated distinct clinical features in the Chinese population. Large-scale population-based studies are needed to further evaluate these findings.

## 1. Introduction

Inflammatory bowel disease (IBD) is a group of chronic gastrointestinal disorders, which is thought to be developed from the complex interactions between genetic predisposition, environmental factors, and a dysregulated immune response [1,2]. Differences in the prevalence and incidence of IBD among various ethnic populations and geographic regions have been broadly documented [3]. The highest prevalence of IBD is reported in the North American and European areas, affecting up to 3.5 million individuals; whereas in Asian and African countries, it’s considered to be relatively rare [4]. Recent systematic reviews indicate the disease burden of IBD is increasing globally, including in the traditional low-prevalent Asian areas, such as Malaysia, Singapore, and Japan [5,6].

In China, the rising occurrence of IBD cases has been reported in clinical studies, but epidemiological studies estimating the incidence and prevalence rate of IBD are not abundant [7,8]. As a result, the nation-wide disease burden of IBD in China is difficult to estimate and this has limited the research community’s ability to understand and forecast the worldwide epidemiological framework of IBD [9]. Although the incidence and prevalence rate of IBD in China is believed to be lower than that in developed countries, when taking into account the fact that China has a large population (more than one billion), in conjunction with expanding urbanization and westernization, the number of IBD cases in China might not be much less than the number of cases in the Western world. Thus, the global disease burden of IBD might be underestimated, due to a lack of epidemiological data from China.

Moreover, reports on the clinical characteristics and presentations of IBD patients from different ethnicities are also inconsistent [10,11]. Significant differences in the phenotypic manifestations have been reported between the Chinese and Caucasian patients [12,13]. Chinese patients were found to have a smaller number of disease lesions in their colon and rectum, and a lower rate of more severe manifestations, than Caucasian patients [12,13]. However, these clinical studies have not generally been conducted with a large enough sample size to present the distinct clinical features in Chinese IBD patients. Gaining a better understanding of the phenotypic features among different ethnicities will probably provide some clues for investigating the environmental and genetic determinants of IBD.

The rising occurrence of IBD cases and possible differences in the clinical characteristics among different races, indicate that the clinical experience in developed countries is unlikely to be applicable to China, which highlights the need to carry out further studies to investigate the epidemiological and clinical characteristics of Chinese patients. To provide the international research community with an overview of the disease burden and the key clinical features of IBD in China, we conducted this systematic review and meta-analysis to estimate the crude incidence and prevalence rate, and to summarize the clinical characteristics, by using the published data from both English and Chinese literature.

## 2. Methods

### 2.1. Literature Search and Selection Criteria

Two English databases (Medline and EMBASE) and two Chinese databases (China National Knowledge Internet and Wanfang digital database) were systematically searched from inception to March 2016, to identify relevant studies by using the search terms related to inflammatory bowel disease (Supplementary Table S1). The identified publications were reviewed independently by two authors (Xue Li and Peige Song), based on the pre-defined inclusion and exclusion criteria. Both population- and hospital-based studies of IBD in the Chinese population were included. The included studies either reported the incidence or prevalence rate, or the clinical characteristics, of IBD. We only included original studies. Thus, systematic reviews and meta-analyses were excluded. Studies on Chinese migrants were not included. All included studies had a clear case ascertainment, based on appropriate clinical, endoscopic, histopathological, and radiological findings.

### 2.2. Data Extraction and Managemen

A standardized data extraction form was designed to capture all relevant study-specific information required for analysis. For each study, the following data was extracted independently by two authors (Xue Li and Peige Song), for analysis: (i) identification details: the first author, publication year, study location, study period, study design, diagnosis criteria, and sample source; (ii) epidemiological characteristics: sample size, number of IBD cases (male/female), reported incidence, and prevalence rate; (iii) clinical characteristics: presenting symptoms, extra-intestinal manifestations, the disease activity, and classifications. Data on the disease classification were extracted based on the Montreal classification criteria, and patients were grouped based on the age at diagnosis, disease location, behaviour, and severity [14]. Double extracted data were checked by a third author (J.L.) and any discrepancy was resolved by discussion.

### 2.3. Data Analysis

The incidence rate of IBD was defined as the number of cases over the total person-years at risk in the study population. The summary crude incidence rate (per 100,000 person-years) with 95% confidence intervals (95% CI), was estimated by using either the random-effect or fixed-effect model. The heterogeneity between studies was estimated by using a Q test and *I*^2^. When substantial heterogeneity was observed (*p* < 0.10 or *I*^2^ > 75%), random-effect models were used for meta-analysis; otherwise, fixed-effect models were applied. Studies were weighted by person-years, which considered both the population size and the duration of observation. Since only a very small number of studies reported data on the prevalence rate of IBD, a meta-analysis was not applicable, and thus, we performed a descriptive analysis. The constituent ratios with 95% CI were calculated for each clinical phenotype and classification. All statistical analyses were conducted by using Stata software (Release 14, Stata Corp L.P., College Station, TX, USA). The reported incidence rate of IBD for different provincial areas was mapped by geographic regions. If multiple studies reported incidence rates for the same location, we used the one which had been conducted most recently. If one study reported incidence rates for multiple instinct locations, the study was split according to the corresponding locations. Geographic maps were created by ArcGIS 10.1 software (Environmental Systems Research Institute, Redlands, CA, USA), based on the open-access “China” shapefile provided by the GADM database of Global Administrative Areas (version 2.8, www.gadm.org/). 

## 3. Results

**Figure 1:**
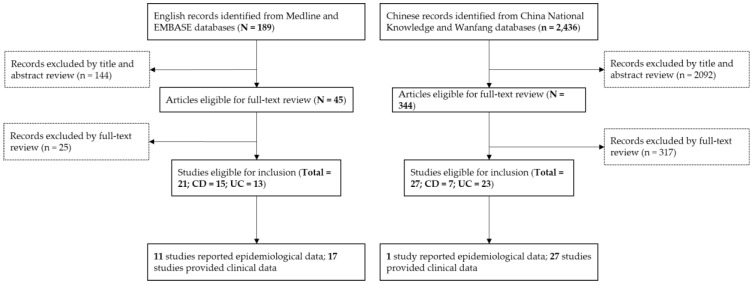
PRISMA flow diagram for literature review.

The search strategy retrieved 2436 unique Chinese publications and 189 unique English publications. Of these, 2092 Chinese papers and 144 English papers were excluded using the title and abstract review, leaving 344 Chinese papers and 45 English papers for full text review. A total of 27 Chinese publications and 21 English publications were finally included in the study, after the full text review (Figure 1). Of these, seven studies reported the incidence rate of Crohn’s disease (CD) and three studies reported the prevalence rate of CD. For ulcerative colitis (UC), seven studies reported the incidence rate and three studies reported the prevalence rate. Four studies reported the prevalence rate of IBD with a combination of CD and UC. The clinical characteristics of CD were reported in 18 studies, with a median number of 69 CD cases (range: 15–515). The clinical characteristics of UC were reported in 35 studies, with a median number of 189 UC cases (range: 35–3922). The characteristics of the included studies can be seen in Supplementary Table S2.

**Figure 2:**
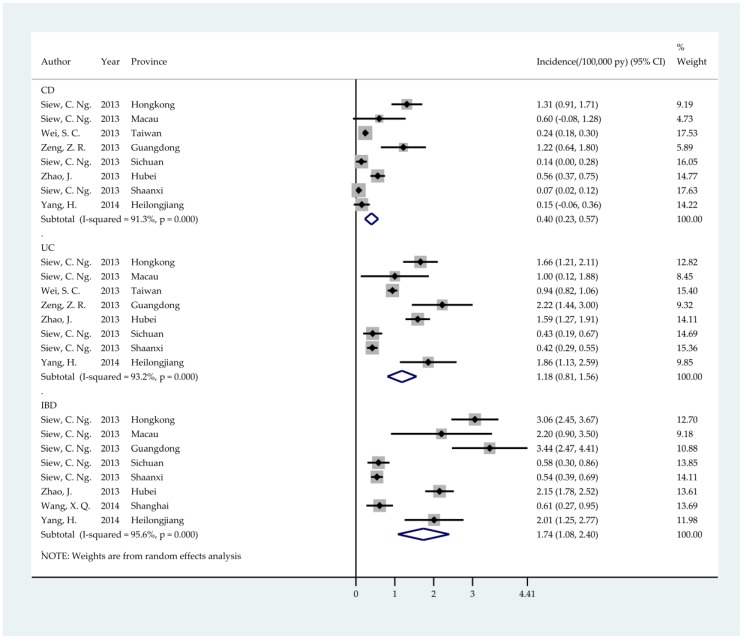
Forest plot for the meta-analysis of the incidence rate of inflammatory bowel disease.

**Figure 3:**
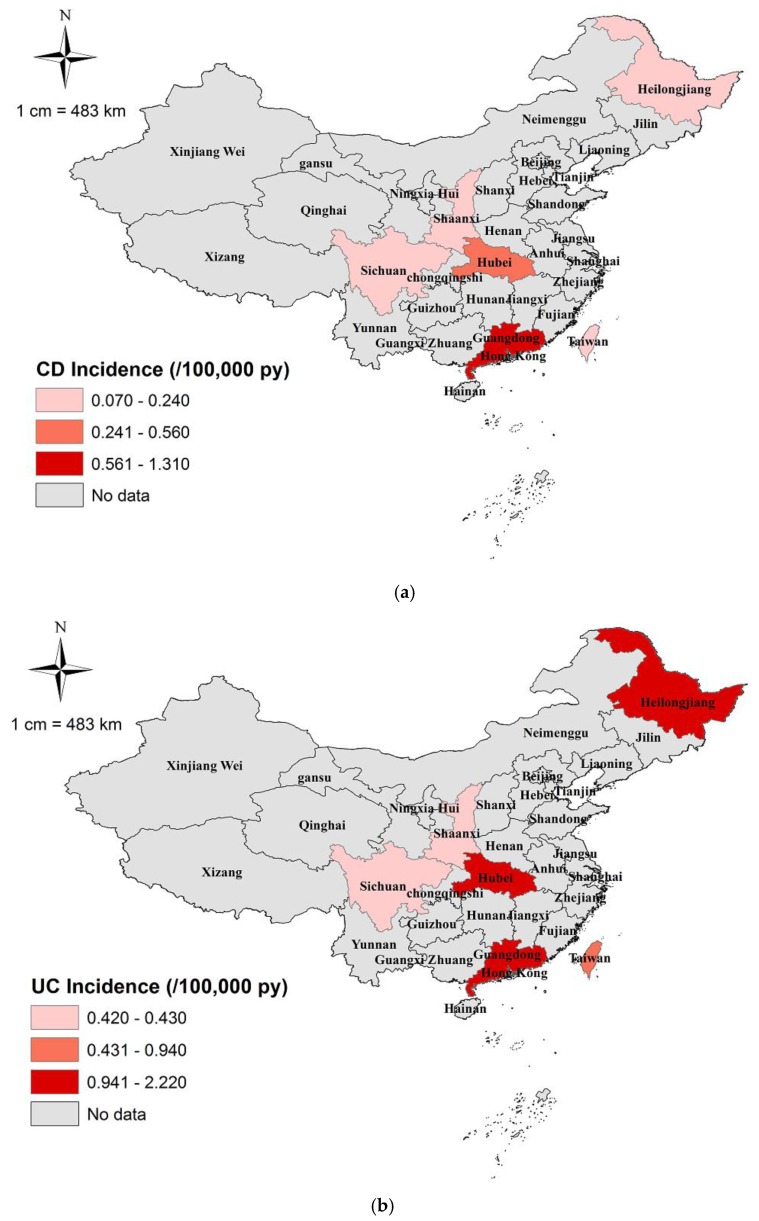

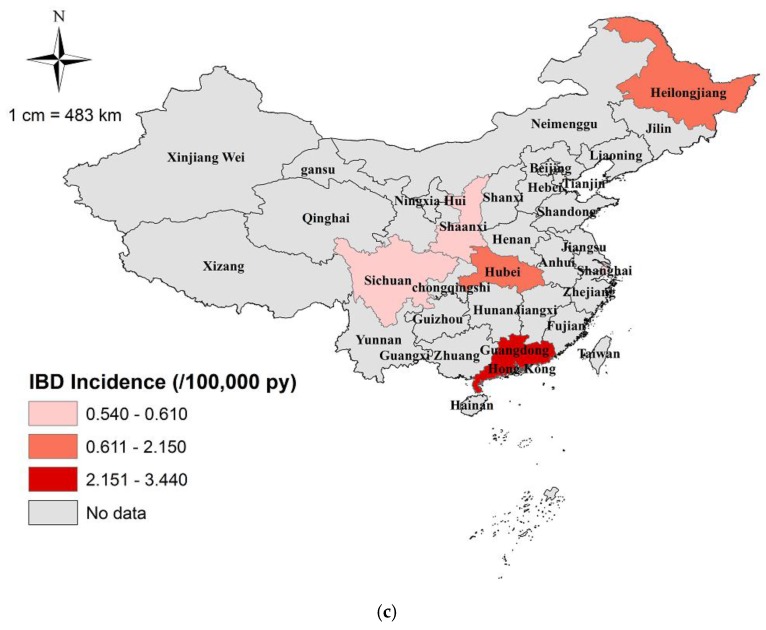
National map of the incidence rate of inflammatory bowel disease: (**a**) Crohn’s disease (CD); (**b**) ulcerative colitis (UC); (**c**) inflammatory bowel disease (CD and UC).

The summary incidence rate of IBD pooled by meta-analysis is 1.74 (95% CI: 1.08; 2.40) per 100,000 person years, and the corresponding incidence rates of CD and UC are 0.40 (95% CI: 0.23; 0.57) and 1.18 (95% CI: 0.81; 1.56) per 100,000 person years, respectively (Figure 2). The incidence rates varied by region, with the incidence rates of CD ranging from 0.07 to 1.31 (median: 0.40) per 100,000 person years (Figure 3) and the incidence rates of UC ranging from 0.42 to 2.22 (median: 1.30) per 100,000 person years (Figure 3). The prevalence of IBD was reported in three studies, in which the prevalence rates of CD and UC in Hong Kong were 2.70 and 5.12 per 100,000 persons, respectively. The prevalence rate of IBD in Taiwan was 5.9 per 100,000 persons.

The clinical characteristics of 2283 CD cases are shown in Table 1. The median age of CD patients was 37.30 years. When considering the age breakdown of CD cases, 13.9% (95% CI: 12.2%–15.7%) of patients were diagnosed at the age of 16 or younger (A1), 64.2% (95% CI: 61.8%–66.7%) were between 17–40 years old (A2), and 21.9% (95% CI: 19.7%–24.0%) were older than 40 years (A3). The median sex ratio (male to female) of CD cases was 1.65 (range: 1.25–2.45), and the pooled sex ratio by meta-analysis was 1.6 (95% CI: 1.5–1.8). Phenotypic classifications showed that 30.3% (95% CI: 28.3%–32.2%) of CD patients had disease lesions in the terminal ileum (L1), 31.2% (95% CI: 29.2%–33.1%) in the colon (L2), 35.4% (95% CI: 33.4%–37.5%) in the ileocolon (L3), and 2.2% (95% CI: 1.6%–2.8%) in the upper gastro-intestine (L4). When considering the behaviour of CD, the structuring disease (B2) and penetrating disease (B3) were presented in 29.0% (95% CI: 26.5%–31.6%) and 19.2% (95% CI: 17.0%–21.4%) of the patients, respectively, and the majority (44.1%; 95% CI: 41.3%–46.9%) had a non-stricturing and non-penetrating disease (B1). The predominant clinical symptoms in CD patients were abdominal pain (79.4%; 95% CI: 76.8%–82.1%), diarrhoea (54.2%; 95% CI: 51.0%–57.4%), weight loss (44.4%; 95% CI: 41.2%–47.6%), fever (32.0%; 95% CI: 29.0%–35.0%), anaemia (24.8%; 95% CI: 22.0%–27.6%), bloody stool (16.0%; 95% CI: 13.7%–18.4%), and haemorrhage (15.0%; 95% CI: 12.7%–17.4%). The overall prevalence of extra-intestinal manifestation was 19.9% (95% CI: 17.4%–22.4%) and the most common extra-intestinal manifestations were observed in joints (7.1%; 95% CI: 5.5%–8.7%), the mouth (5.8%; 95% CI: 4.3%–7.2%), skin (2.9%; 95% CI: 1.8%–3.9%), and biliary (2.6%; 95% CI: 1.6%–3.5%).

The clinical characteristics of 17,958 UC cases are presented in Table 2. The median age for UC cases was 43.10 years. When focusing on the age breakdown of UC cases, 2.1% (95% CI: 1.8%–2.4%) were diagnosed at the age of 16 or younger (A1), 34.0% (95% CI: 33.0%–35.0%) were between 17–40 years old (A2), and 63.9% (95% CI: 62.9%–64.9%) were older than 40 years (A3). The median sex ratio (male to female) was 1.28 (range: 0.84–2.74), and the pooled sex ratio by meta-analysis was 1.29 (95% CI: 1.21–1.38). The summary data showed that 26.7% (95% CI: 26.1%–27.4%) of UC cases had proctitis, 48.3% (95% CI: 47.5%–49.0%) had proctosigmoiditis, 16.1% (95% CI: 15.6%–16.7%) had pancolitis, and 8.9% (95% CI: 8.4%–9.3%) had extensive colitis. When considering the severity of UC, 30.3% (95% CI: 29.4%–31.1%) of patients had a mild degree of disease, 46.3% (95% CI: 45.3%–47.2%) a moderate degree, and 23.5% (95% CI: 22.7%–24.3%) a severe degree. When studying the clinical symptoms, diarrhoea (83.7%; 95% CI: 83.0%–84.3%), abdominal pain (64.9%; 95% CI: 64.1%–65.8%), bloody stool (53.1%; 95% CI: 52.1%–54.0%), mucus (28.8%; 95% CI: 28.0%–29.6%), and weight loss (28.4%; 95% CI: 27.7%–29.3%), were most frequently presented in UC patients. The proportion of UC patients with extra-intestinal manifestations was 10.9% (95% CI: 10.2%–11.6%), with 5.4% (95% CI: 4.8%–5.9%) in the joints, 2.1% (95% CI: 1.8%–2.5%) in the mouth, 1.5% (95% CI: 1.2%–1.8%) in the skin, and 1.0% (95% CI: 0.8%–1.2%) in the eyes.

## 4. Discussion

In this study, we performed an up-to-date systematic review of the epidemiological and phenotypic characteristics of IBD, to better understand the disease status in China. This review estimated the crude summary incidence and prevalence of IBD in the Chinese population, which will help researchers to evaluate the global effects of IBD. By collating the clinical features of 2283 CD cases and 17,958 UC cases, we provided a summary of the phenotypic characteristics of Chinese IBD patients. The clinical differences between patients from China and other developed countries are discussed. We believe that this review will be a helpful resource for researchers to understand the differences in the epidemiology and clinical characteristics of IBD in the East and West.

In our study, the estimated that the incidence rate was 0.40 per 100,000 person years for CD, 1.18 per 100,000 person years for UC, and 1.74 per 100,000 person years for IBD, which are much lower than those in European and American countries [15,16], as well as other Asian countries, like Japan, South Korea, and Singapore [6,17]. Most studies from western countries have reported a female predominance in the incidence of CD and no significant difference in the sex-specific incidence rate of UC [18,19]. However, our study observed a male predominance in both CD and UC, and this preponderance of male patients has also been consistently reported in other Asian countries [20,21]. Additionally, our study identified a geographic aggregation in IBD incidence. It has been reported that the total incidence of IBD in the northern and western Europe is several times higher than that in the south and east, with an incidence rate ratio (IRR) of 1.90 for CD and 2.10 for UC [22,23]. However, in China, a higher IBD incidence was reported in the economically well-developed southeast areas, indicating an inverse geographic gradient. A previously published systematic review reported an extrapolated incidence rate of 0.85 per 100,000 person years, based on data from three southeast cities, which was relatively higher than the nation-wide incidence rate estimated in this study [9].

The male predominance and apparent variations in IBD incidence among different provincial areas (very large heterogeneity: *I*^2^ = 95.6%) might be primarily explained by the differences in environmental determinants derived from biological, social, and economic exposures [24,25]. Smoking is one of the most consistently studied environmental factors of IBD. The global adult tobacco survey has shown that the prevalence of smoking in men (50%) is much higher than that in women (11%) [26], which might have contributed to the male predominance of developing CD by influencing the intestinal microbiota [27]. However, a paradox exists for the smoking hypothesis regarding the male predominance of UC, as the high smoking exposure in men is expected to decrease the risk of developing UC [28]. Alternatively, the influence of hormones on the brain-gut-microbiota axis is hypothesized for the sex difference of IBD development, but the mechanism underlying this complex pathophysiology is still not completely understood [29]. The large heterogeneity of IBD incidence rates across different geographic areas is likely attributed to the regional unevenness in socioeconomic development. For example, in the well-developed southeast areas, such as Guangzhou and Hong Kong, which have shifted to a more “westernized” lifestyle, the disease risk was detected as being higher than in other regions. This finding is in accordance with the phenomenon across the world, where the lifestyle in developed countries is believed to impair the natural patterns of microbial colonization of the human gut [30]. Furthermore, the advances in healthcare infrastructure, or the epidemiological surveillance in more developed areas, can also accelerate the detection of IBD cases.

Concerning the phenotypic and clinical characteristics of CD, most of the Chinese patients were diagnosed between 16–40 years, with non-stricturing and non-penetrating disease, varied disease locations, less extra-intestinal manifestations, and typical presenting symptoms. A bimodal distribution of age at the diagnosis of CD has been documented in developed areas, with the first peak at 20–30 years old and a second at 60–70 years old [31,32]. Similarly, in the Chinese population, most of the CD cases were observed in the 17–40 age group (64.2%) and the older than 40 years group came second (21.9%). Compared to the developed areas, where approximately 25%–30% of CD occurred in childhood and adolescence [33], China had a relatively lower proportion (13.9%) of pediatric CD cases. When considering the disease behavior, the Chinese CD patients had less stricturing (29.0%) or penetrating disease (19.2%), when comparing the findings from France and Belgian cohorts, in which up to 70% of CD patients developed either stricturing or penetrating disease [34,35]. Data on the disease locations in developed countries are relatively homogeneous with the preponderance of colonic involvement and rare GI inflammation. As an example, a recent Norwegian cohort found that 27% of CD patients had a disease lesion in their ileum (L1), 48% in their colon (L2), 23% in their ileocolon (L3), and 2% in their Upper GI (L4) [36]. In comparison, the Chinese patients had a similar proportion of GI involvement (L4: 2.2%), but a relatively lower rate of colonic disease (L2: 31.2%). When investigating the extra-intestinal manifestations (EIMs), it’s estimated that EIMs presented in around 19.9% of Chinese patients, but up to 20%–40% of European patients [37]. The most common EIMs presented as arthritis in both Chinese and European patients, which occurred in 7.1% of Chinese cases and up to 20%–30% of European cases [37,38]. As to the clinical symptoms, there is no difference between China and developed countries, with the top clinical complaints consistently being associated with abdominal pain, diarrhoea, and weight loss [39,40].

Regarding the clinical characteristics of UC, Chinese cases were featured with later disease diagnosis, a more severe disease course, more segmental lesions, and less extra-intestinal manifestations. Data from developed countries showed that UC was most likely to affect young adults of 15–30 years old [31]. However, the age distribution of Chinese UC patients suggested the older people were the most predisposed population, with 63.9% of UC cases diagnosed when the patient was older than 40 years. Patients diagnosed at the age of 16 or younger only accounted for 2.1% in China, while the corresponding proportion in developed countries was up to 20% [33]. When comparing the disease severity, our study suggested that approximately 70% of Chinese UC patients had a moderate or severe disease course, while reports from developed countries indicated that 60% of UC patients had a mild degree disease, and the remaining 40% had a moderate and severe disease [41,42]. The high proportion of severe disease in Chinese UC patients might be overestimated, given the fact that the clinical data in our study came from hospital-based studies and most of the patients with mild degree disease might not have been admitted to hospitals. When comparing the disease locations, patients in developed countries were more likely suffer from an extensive spread of the disease, with the proportion of pancolitis being 24% and extensive colitis being 33% [43], while the corresponding proportion of Chinese patients was 16.1% and 8.9%, respectively. When considering EIMs, the overall prevalence of EIMs in Chinese UC patients was 10.9%, which was much lower than the rate of 40% reported in western countries [38,44,45]. Similar to CD, there was no substantial difference in the clinical symptoms between patients from China and other western countries, with the main complaints being diarrheoa, abdominal pain, and bloody stool [39,40].

## 5. Conclusions

In summary, by synthesizing the published data, our study provided an estimated disease burden of IBD in the Chinese population. Our study indicated that the clinical features of IBD in China were different from those in developed countries, in terms of age and sex distribution, disease location and severity, and the prevalence of extra-intestinal manifestations. Since most of the current studies in China were based in hospitals, our study may introduce some bias, which can potentially result in the underestimation of the true incidence, and an overestimation of disease severity. Our study provided information in relation to the disease incidence, prevalence, and clinical characteristics, and large-scale population-based studies are needed to further evaluate these findings.

## Figures and Tables

**Table 1:** The phenotypic and clinical characteristics of Crohn’s disease.

CD Phenotype	No. of Cases	Proportion (%)	95% CI
**Age at diagnosis (*n* = 1464)**			
A1 (≤16 years)	204	13.9	12.2 to 15.7
A2 (17–40 years)	940	64.2	61.8 to 66.7
A3 (<40 years)	320	21.9	19.7 to 24.0
**Location (*n* = 2153)**			
L1 (Terminal ileum)	652	30.3	28.3 to 32.2
L2 (Colon)	671	31.2	29.2 to 33.1
L3 (Ileocolon)	763	35.4	33.4 to 37.5
L4 (Upper GI)	47	2.2	1.6 to 2.8
L1 + L4 (Terminal ileum + Upper GI)	8	0.4	0.1 to 0.6
L2 + L4 (Colon + Upper GI)	1	0.1	0.0 to 0.2
L3 + L4 (Ileocolon + Upper GI)	11	0.5	0.2 to 0.8
**Behaviour (*n* = 1221)**			
B1 (Non-stricturing + non-penetrating)	538	44.1	41.3 to 46.9
B2 (Stricturing)	355	29.0	26.5 to 31.6
B3 (Penetrating)	234	19.2	17.0 to 21.4
B1p (Non-stricturing + non-penetrating + perianal)	59	4.8	3.6 to 6.0
B2p (Stricturing + perianal)	10	0.8	0.3 to 1.3
B3p (Penetrating + perianal)	25	2.1	1.3 to 2.8
**Clinical symptoms (*n* = 924)**			
Abdominal pain	734	79.4	76.8 to 82.1
Diarrhoea	501	54.2	51.0 to 57.4
Weight loss	410	44.4	41.2 to 47.6
Fever	296	32.0	29.0 to 35.0
Anaemia	229	24.8	22.0 to 27.6
Bloody stool	148	16.0	13.7 to 18.4
Haemorrhage	139	15.0	12.7 to 17.3
Constipation	71	7.7	6.0 to 9.4
**Extra-intestinal manifestations (*n* = 979)**			
Joint	69	7.1	5.5 to 8.7
Mouth	56	5.8	4.3 to 7.2
Skin	28	2.9	1.8 to 3.9
Biliary	25	2.6	1.6 to 3.5
Eyes	16	1.6	0.8 to 2.4
Others	25	2.6	1.6 to 3.5

**Table 2:** The phenotypic and clinical characteristics of ulcerative colitis.

UC Phenotype	No. of Cases	Proportion (%)	95% CI
**Age at diagnosis (N = 8895)**			
A1 (≤16 years)	190	2.1	1.8 to 2.4
A2 (17–40 years)	3025	34.0	33.0 to 35.0
A3 (<40 years)	5680	63.9	62.9 to 64.9
**Location (*n* = 17,371)**			
Proctitis	4643	26.7	26.1 to 27.4
Proctosigmoiditis	8384	48.3	47.5 to 49.0
Pancolitis	2798	16.1	15.6 to 16.7
Extensive colitis	1546	8.9	8.4 to 9.3
**Severity (*n* = 11,389)**			
Mild	3447	30.3	29.4 to 31.1
Moderate	5268	46.3	45.3 to 47.2
Severe	2674	23.5	22.7 to 24.3
**Clinical symptoms (*n* = 11,305)**			
Diarrhoea	9457	83.7	83.0 to 84.3
Abdominal pain	7340	64.9	64.1 to 65.8
Bloody stool	5997	53.1	52.1 to 54.0
Weight loss	3215	28.4	27.7 to 29.3
Mucus	3254	28.8	28.0 to 29.6
Fever	2054	18.2	17.5 to 18.9
Anaemia	1512	13.4	12.8 to 14.0
Constipation	654	5.8	5.4 to 6.2
Haemorrhage	257	2.3	2.0 to 2.6
**Extra-intestinal manifestations (*n* = 6886)**			
Joint	368	5.4	4.8 to 5.9
Mouth	146	2.1	1.8 to 2.5
Skin	101	1.5	1.2 to 1.8
Eyes	68	1.0	0.8 to 1.2
Biliary	4	0.1	0.0 to 0.1
Others	65	1.0	0.7 to 1.2

## Supplementary Materials

The following are available online at www.mdpi.com/1660-4601/14/3/238/s1, Table S1: Keywords and search strategies, Table S2: The characteristics of included studies.
