# Supplementary material for: The Disease Burden and Clinical Characteristics of Inflammatory Bowel Disease in the Chinese Population: A Systematic Review and Meta-Analysis
Source: Int J Environ Res Public Health. 2017 Feb 28;14(3):238. doi: 10.3390/ijerph14030238 (PMC5369074; doi:10.3390/ijerph14030238)
Supplement: Supplementary file 1 [file ijerph-14-00238-s001.pdf]

Supplementary Materials: The Disease Burden and Clinical Characteristics of Inflammatory Bowel Disease in Chinese Population: A Systematic Review and Meta-Analysis

Xue Li, Peige Song, Jun Li, Yuchang Tao, Guowei Li, Xiumin Li * and Zengli Yu *

Table S1. Keywords and search strategies.

Database	Access Date	Search Terms	Search Method
CNKI	March 2016	(SU % '炎性肠病' + '克罗恩' + '溃疡性结肠炎' + 'IBD' OR TI % '炎性肠病' + '克罗恩' + '溃疡性结肠炎' + 'IBD' OR KY % '炎性肠病' + '克罗恩' + '溃疡性结肠炎' + 'IBD' OR AB % '炎性肠病' + '克罗恩' + '溃疡性结肠炎' + 'IBD') AND (SU % '患病率' + '发病率' + '现患率' + '罹患' + '流行' + '调查' + '现况'+ '病例' + '临床' + '分析' OR TI % '患病率' + '发病率' + '现患率' + '罹患' + '流行' + '调查' + '现况'+ '病例' + '临床' + '分析' OR KY % '患病率' + '发病率' + '现患率' + '罹患' + '流行' + '调查' + '现况'+ '病例' + '临床' + '分析' OR AB % '患病率' + '发病率' + '现患率' + '罹患' + '流行' + '调查' + '现况'+ '病例' + '临床' + '分析')	Comprehensive search: subject, title, keywords and abstract
Wanfang	March 2016	(“炎性肠病” OR “克罗恩” OR “溃疡性结肠炎” OR “IBD”) AND (“患病率” OR “发病率” OR “现患率” OR “罹患” OR “流行” OR “调查” OR “现况” OR “病例” OR “临床” OR “分析”)	Comprehensive search: subject (including title, keywords and abstract)
Medline & EMBASE	March 2016	(Inflammatory bowel disease OR Crohn disease OR ulcerative colitis) AND (prevalence OR incidence OR cross-sectional OR cohort OR survey) AND (China OR Chinese)	MeSH headings & keywords

Table S2. The characteristics of included studies.

Author	Publish Year	Location	Territorial Level	Study Period	Sample Source	No. of Cases	Data provided		
							Incidence	Prevalence	Clinical feature
English literature									
IBD Working Group	2006	11 provincial areas	National wide	1990–2003	Hospital-based	515 CD	n/a	n/a	Yes
Cao, Q.	2006	Hangzhou	City	1994–2004	Hospital-based	62 CD; 317 UC	n/a	n/a	Yes
Chen, Y. C.	2008	Taiwan	Provincial	2004	National Health Insurance Database	1209 IBD	n/a	$5.9/10^5$	n/a
Chow, D. K.	2009	Hong Kong	Provincial	1985–2006	Hospital-based	172 UC	$2.1/10^5$	n/a	n/a
Gong, W.	2012	5 provincial areas	National wide	1998–2009	Hospital-based	3922 UC	n/a	n/a	Yes
Huang, M. L.	2014	Shanghai	City	2001–2012	Hospital-based	317 CD	n/a	n/a	Yes
Jiang, L.	2007	Wuhan	City	2004	Hospital-based	177 UC	n/a	n/a	Yes
Jiang, L.	2006	Wuhan	City	1990–2003	Hospital-based	63 CD; 389 UC	n/a	n/a	Yes
Leong, R. W.	2004	Hong Kong	Provincial	2001	Hospital-based	80 CD	$3.0/10^5$	n/a	Yes
Lok, K. H.	2008	Hong Kong	Provincial	1990–2006	Hospital-based	73 UC	$0.73/10^5$	$5.12/10^5$	Yes
Lok, K. H.	2007	Hong Kong	Provincial	1991–2006	Hospital-based	27 CD	$0.25/10^5$	$2.70/10^5$	Yes
Siew C. Ng.	2013	Hong Kong	Provincial	2011–2012	Hospital-based	42 CD; 56 UC	CD:$1.31/10^5$; UC: $1.66/10^5$	n/a	n/a
		Macau	City	2011–2012	Hospital-based	3 CD; 8 UC	CD:$0.60/10^5$; UC: $1.00/10^5$	n/a	n/a
		Guangzhou	City	2011–2012	Hospital-based	17 CD; 31 UC	CD:$1.22/10^5$; UC: $2.22/10^5$	n/a	n/a
		Xi'an	City	2011–2012	Hospital-based	6 CD; 42 UC	CD:$0.07/10^5$; UC: $0.42/10^5$	n/a	n/a
		Chengdu	City	2011–2012	Hospital-based	4 CD; 12 UC	CD:$0.14/10^5$; UC: $0.43/10^5$	n/a	n/a

Wang, X. Q.	2013	Shanghai	City	2000–2010	Hospital-based	82 CD; 73 UC	IBD: $0.61/10^5$	n/a	Yes
Xiang, Z.	2013	Hangzhou	City	2008–2012	Hospital-based	229 CD	n/a	n/a	Yes
Yang, H.	2014	Daqing	City	2012–2013	Hospital-based	2 CD 25 UC	CD: $0.14/10^5$ UC:$1.86/10^5$	n/a	Yes
Yi, F.	2013	Wuhan	City	2006–2011	Hospital-based	189 UC	n/a	n/a	Yes
Zeng, Z.	2013	Zhongshan	City	2011–2012	Hospital-based	17 CD 31 UC	CD:$1.22/10^5$ UC:$2.22/10^5$	n/a	Yes
Zhao, J.	2013	Wuhan	City	2010	Hospital-based	34 CD 97 UC	CD:$0.06/10^5$ UC:$0.16/10^5$	n/a	Yes
Wei, S.C.	2013	Taiwan	Provincial	1998–2008	National Health Insurance Database	385 CD 1206 UC	CD:$0.24/10^5$ UC:$0.94/10^5$	n/a	n/a
Zhou, F.	2010	Wuhan	City	2004–2009	Hospital-based	54 CD	n/a	n/a	Yes
Luo, C. H.	2011	Beijing	City	1985–2004	Hospital-based	85 CD	n/a	n/a	Yes
Chinese literature									
Gao, Y.	2006	Zunyi	City	1995–2004	Hospital-based	15 CD	n/a	n/a	Yes
Chen, C. H.	2006	Hohhot	City	1995–2005	Hospital-based	640 UC	n/a	n/a	Yes
Yan, W.	2007	Beijing	City	1978–2006	Hospital-based	327 UC	n/a	n/a	Yes
Shen, H. Q.	2003	Beijing	City	1992–2002	Hospital-based	27 UC	n/a	n/a	Yes
Lin, X. L.	2011	Guangzhou	City	1999–2009	Hospital-based	227 UC	n/a	n/a	Yes
Zhu, Z. H.	2013	Zhongshan	City	2011–2012	Hospital-based	31 UC	$2.22/10^5$	n/a	Yes
Gao, X.	2005	Zhongshan	City	1990–2003	Hospital-based	89 CD; 161 UC	n/a	n/a	Yes
Gao, F.	2007	Urumchi	City	2000–2006	Hospital-based	699 UC	n/a	n/a	Yes
Ju, W. W.	2007	Liaodong	City	1995–2005	Hospital-based	69 CD; 131 UC	n/a	n/a	Yes
Chen, Y. Y.	2007	Hainan	City	1996–2006	Hospital-based	105 UC	n/a	n/a	Yes
Hu, H. Y.	2008	Beijing	City	1990–2006	Hospital-based	254 UC	n/a	n/a	Yes
Luo, Y. Y.	2007	Hangzhou	City	1992–2005	Hospital-based	114 CD	n/a	n/a	Yes
Gan, H. T.	2000	Chengdu	City	1989–1998	Hospital-based	55 CD	n/a	n/a	Yes
Wang, Y. F.	2005	Chengdu	City	1990–2003	Hospital-based	357 UC	n/a	n/a	Yes
Wang, X. D.	2006	Beijing	City	1984–2004	Hospital-based	805 UC	n/a	n/a	Yes
Wang, X. Y.	2011	Beijing	City	2009–2011	Hospital-based	261 UC	n/a	n/a	Yes
Wang, N.	2012	Xi'an	City	2008–2011	Hospital-based	360 UC	n/a	n/a	Yes
Yang, Y.	2006	Nanjing	City	2000–2005	Hospital-based	85 CD	n/a	n/a	Yes

Yang, L.	2013	Changsha	City	2003–2012	Hospital-based	28 CD; 83 UC	n/a	n/a	Yes
Xu, Z. J.	2009	Nanjing	City	2001–2008	Hospital-based	68 UC	n/a	n/a	Yes
Zhang, Y.	2010	Dali	City	2001–2008	Hospital-based	43 UC	n/a	n/a	Yes
Zhang, J. M.	2014	Shenyang	City	2000–2013	Hospital-based	260 UC	n/a	n/a	Yes
Jiang, L.	2012	Wuhan	City	1991–2010	Hospital-based	223 UC	n/a	n/a	Yes
Yao, J.	2010	Jinzhou	City	1999–2009	Hospital-based	31 UC	n/a	n/a	Yes
Liu, Y. D.	2015	Tianjin	City	2008–2013	Hospital-based	1593 UC	n/a	n/a	Yes
Liu, Y.	2016	Kunming	City	2007–2013	Hospital-based	459 UC	n/a	n/a	Yes
IBD Working Group	2006	11 provincial areas	National wide	1990-2003	Hospital-based	1775 UC	n/a	n/a	Yes

© 2017 by the authors; licensee MDPI, Basel, Switzerland. This article is an open access article distributed under the terms and conditions of the Creative Commons by Attribution (CC-BY) license (http://creativecommons.org/licenses/by/4.0/).
